# On Painful and Permanent Spasmodic Stricture of the Sphincter Ani, with or without Fissure

**Published:** 1828-12

**Authors:** 


### THE LONDON
### Medical and Physical Journal.

N°. 358, VOL. LX.] DECEMBER, 1828. [N°. 30, *New Series.*

For many fortunate discoveries in medicine, and for the detection of numerous errors, the world is indebted to the rapid circulation of Monthly Journals; and there never existed any work, to which the Faculty, in Europe and America, were under deeper obligations, than to the *Medical and Physical Journal of London,* now forming a long, but an invaluable, series.—RUSH.

### ORIGINAL PAPERS,

AND

### CASES OBTAINED FROM PUBLIC INSTITUTIONS AND OTHER AUTHENTIC SOURCES.

### STRICTURE OF THE SPHINCTER ANI.

*On Painful and Permanent Spasmodic Stricture of the Sphincter Ani, with or without Fissure.* By A CORRESPONDENT IN PARIS.

I PERCEIVE, in Dr. JOHNSON'S review of a Treatise on Stricture of the Rectum, the following remarks: "A *peculiar constriction of the anus* is sometimes the cause of stricture. Mr. S. has rarely found this unattended with obstruction very high up in the rectum. In females, an occasional and afflicting source of stricture is an enlarged and tender condition of the uterus, of which we have a melancholy case under our care. *Every time that a motion is produced by nature or art, the sufferings are dreadful,* and can only be allayed by the introduction of laudanum into the rectum. *In these unhappy cases, little or nothing can be done by surgery.* The only means of mitigating the pains are by emollient injections, to bring away the feces in a soft state; and afterwards throwing up an anodyne."

To say nothing of the manifestly incredible statement of Mr. Salmon, that constriction of the anus is *rarely* found without obstruction very high up in the rectum, the supposed internal disease which is thus asserted to be beyond the reach of surgery, I presume to be one which is known to continental surgeons under the title of spasmodic constriction of the sphincter, accompanied most commonly by fissure within the gut, and is *cured* by cutting freely through all the fibres of the sphincter in one or more parts, avoiding the anterior and posterior, on account of the important organs in front of the one, and the blood-vessels of the other.

The symptoms of the patient referred to by the reviewer are supposed to arise from an "enlarged and tender condition of the uterus:" so, in cases of the spasmodic affection of the anus, women have ascribed their sufferings to ulcers of the womb; sometimes they have been treated for disease of the liver or spleen; and the cause in others has been referred to syphilis, or impetiginous affection: but all these accessary symptoms disappeared by the free incision of the sphincter.

The uterine affection, with constant desire to void urine, was strongly marked in a patient of the Hôtel Dieu at Paris, on whom I witnessed the operation. The womb was frequently prolapsed and painful; sometimes, in an erect position, she experienced pains which she described as resembling the passage of a fœtus, although not equal in intensity; for, to adopt her own language, they were like a "*petit enfantement.*" She had suffered from hemorrhoids, and violent pain in evacuating the feces, for many years past, and the pains in the interval were lancinating. The introduction of pledgets into the rectum was effected with great difficulty, on account of the constriction, and followed by such intense suffering, that they were necessarily abandoned. When on the operation table in the amphitheatre, the margin of the anus was seen to be completely surrounded by large hemorrhoidal tumors, in the centre of which an internal one appeared; and a fissure existed in the longitudinal direction of the gut. All the tumors were successively removed by the scissors, and an extensive incision was made in the fissure. On the following day, I found her free from pain. Within the space of a fortnight she had recovered her health and cheerfulness; although, for a considerable time past, she had been plunged in the depths of misery and distress.

It is commonly supposed that no traces either of constriction or of the peculiar fissure, are to be found in any author anterior to the time of Sabatier, who speaks of superficial and painful excoriations, narrow and long, within the margin of the anus, which are exceedingly difficult of cure, and remarkable for the pain which they excite. The rhagades and fissures described by authors as concomitants of syphilis, or the same affections noticed by Lemonier in his Treatise on Fistula in Ano, published in 1689, possess none of the characteristic marks of the spasmodic constriction, and the horrible suffering resulting from a fissure which is sometimes scarcely perceptible, and often only to be discovered by the unutterable anguish which follows the introduction of the finger. They are described as little painful ulcers, following the course of the wrinkles of the anus, resembling chaps in the legs and hands; sometimes caused by induration of fecal matter, by dysentery, or by syphilis.

M. Boyer was the first to investigate the nature of this disease, and about twenty years since discovered by accident the proper mode of cure by incision, which he asserts to have successfully employed in upwards of a hundred cases; and his practice has been adopted by the principal surgeons in the French metropolis. His primary intention was no other than to convert a callous fissure into a recent wound, but his success surpassed his expectation. The lancinating pains instantly ceased, and the constriction also. The latter result induced him to make the incision in the case of constriction alone, without fissure: he was equally fortunate. Soon afterwards, having met with patients in whom the fissure existed in the anterior or posterior parts of the rectum, where the use of the knife might be attended with inconvenient results, he determined to make his incisions laterally, taking no account of the fissure, which always disappeared spontaneously after the operation.

It can hardly be necessary to describe the mode of using the scalpel in those cases: it may be sufficient to remind the practitioner, that, as the object is dilatation, the reunion of the lips of the wound should be prevented by the introduction of pledgets into the anus.

Although the essential feature of this affection is the constriction, yet the more frequent accompaniment of the fissure has caused it to be better known by the latter title. M. Boyer gives it this denomination, expressing at the same time his firm opinion that it depends upon the constriction, inasmuch as all the symptoms are felt when the fissure is absent, and the cure of the constriction is invariably followed by that of the fissure.

The account which I believe to be the first in order of publication, appeared in the Dictionnaire des Sciences Medicales, eight years after the successful treatment of the early cases at the Hôpital la Charité, to which M. Boyer is the surgeon. It is, in fact, substantially the same as a later publication of this gentleman, and might be derived from the clinical lectures which, as the professor of surgery, he was in the habit of delivering at the Ecole de Medicine in Paris. Another account was published in the New Dictionnaire de Medicine, in 1824; and it is remarkable that, although some parts are the very words of M. Boyer, yet the work of the latter appeared a year later.*

The characteristics are stated by this gentleman to be excessive pain during the alvine evacuations. The forefinger penetrates with difficulty into the rectum, and is strongly constricted: its introduction is always followed by pain, which is intolerable if strong pressure be made on the fissure, and the patient darts forward to escape the torment which he feels. That which accompanies or follows the alvine discharge is, in general, proportioned to the size and hardness of the excrements; but, although the feces may be liquid as in diarrhœa, the suffering of the patient is still great. Even the evacuation of flatus is productive of pain, and is sometimes impossible. One of the patients was tormented with a desire to discharge flatus to such a degree as to be obliged to wear an elastic tube in the rectum, for the purpose of giving it vent. In many, the symptoms had been preceded by hemorrhoidal tumors, and the removal had not been followed by relief.

It begins insensibly. The evacuation of feces is attended with heat and smarting, which sometimes remains during many hours. Sometimes these symptoms intermit for several days, especially if heating drinks are avoided, and clysters, with frequent cold ablution, be employed. But soon the heat and smarting recurs; the discharge of feces is attended with more pain, and a longer continuance of it. Blood sometimes accompanies the feces; the pains increase. Laxatives, clysters, and cooling regimen, relieve to a certain period, but at length they lose their beneficial effect. The symptoms increase, and require the continual use of purgatives.

The consequence of constipation is a great increase of suffering, which is often compared to the introduction of a red hot iron into the rectum. Some patients are agitated by a sort of general convulsive contraction, or fall into syncope. After the evacuation, the pain is not only acute, but a shooting and pulsation is felt, like that of an inflamed part. In one patient, a slight febrile paroxysm took place after every evacuation.

The pains do not increase in a progressive manner: they intermit, and vary in intensity according to circumstances. Violent exercise, the use of wine and spirits, too much food or of a heating kind, tend to increase them. The influence of regimen is so marked, that patients often confine them-

* Traité des Maladies Chirurgicales, et des Operations qui leur conviennent.

selves to the smallest quantity of food, from a fear of increasing the excrement. In many, the symptoms increase at the menstrual period. One experienced exacerbation of suffering every eighth day.

The most trifling circumstance will exasperate the pain: the act of coughing, voiding urine, or jumping, is sufficient. Some cannot stand erect, others cannot sit.

When the disease has long existed, nervous irritability and emaciation, hypochondriasis, or retention of urine, are frequently superadded to the local symptoms.

The cases treated by M. Boyer so admirably illustrate the disease, that I cannot better conclude this paper than by subjoining them, especially as I do not recollect to have seen them in an English form, and believe them to be generally unknown in Great Britain.

*Cases of Constriction of the Anus, with Fissure.*

Case I.—Marie Aquette, twenty-six years of age, entered the hospital of la Charité, in the month of September, 1809. Two years and a half before this period, she had begun to experience pains in the rectum, which became more acute during the evacuation of fecal matter. Six months afterwards, hemorrhoids appeared, which at first were treated by leeches, and subsequently excised. After the operation the pains increased: they were not constant, and often returned without any apparent cause; sometimes they came on after a fit of coughing or sneezing, or remaining too long either in a recumbent or sitting posture. They also increased at the time of menstruation, and especially on going to stool. Every muscle was then in contraction, and the patient, a prey to unutterable anguish, forcibly grasped every object which surrounded her. When the feces were firm they were small in quantity, commonly tinged with blood, and the pains which they occasioned in the passage was a sense of laceration: when the evacuations were liquid, the smarting was more to be endured, but still great.

Clysters produced some alleviation; but the introduction of the canula was so difficult and painful, that the patient could rarely decide to have recourse to it. The introduction of the finger into the anus (which was frequently examined by the surgeon who usually attended,) constantly produced excessive pain, especially on the right side. After the use of many remedies, a mercurial course was ineffectually tried, to which the patient submitted for nine months, notwithstanding her conviction that her disease could have no connexion with syphilis.

I introduced my finger into the rectum. I felt it strongly pressed. I discovered a fissure on the right side, which, being pressed upon, occasioned horrible pain. Three days afterwards I performed the incision of the sphincter. At the end of fifteen days the stools were free, and without pain. She remained in the hospital two months, and was perfectly cured.

Case II.—The patient speaks :

In the month of May, 1810, I was attacked with violent pain on going to the water closet. I was treated for hemorrhoids. Leeches were applied, and I experienced relief. The violent pains ceased, but during the whole of the summer I suffered in a trifling degree. In the month of December following, I was attacked by a paroxysm of dreadful suffering. The pains became much more violent an hour after having been at the water closet, in which state they remained for five or six consecutive hours. Recourse was then had to leeches, the symptoms being ascribed to hemorrhoids; but on this occasion they produced no effect. The sufferings returned with the same violence at each evacuation. I was then placed on a soft and cooling diet, with hip baths. I was also immersed in warm baths. I passed some days with trifling pain, but every eighth I suffered from paroxysms of increasing violence. Since the month of May last, I have not had a moment's respite. I could not go to stool without experiencing the most agonising pain: it seemed to me that something was lacerating the passage; and, for seven or eight hours after the excretion, I felt a continual pulsation and shooting in the part, with constriction and dryness, as if a red-hot iron had been introduced into it. So violent were the pains that they occasioned fever. The same cooling treatment was continued, but without success. Tents, covered with cerate and opium, were attempted: sometimes they could not be introduced, and, when they were, my sufferings were greatly increased.

M. Boyer was consulted, who ordered injections: they calmed the pain, but did not destroy the evil. He told me that the operation alone could cure me radically. I submitted to it, and in a few days afterwards I went to stool almost without suffering, and now I experience no pain.

Case III.—A needle-woman, thirty-six years of age, felt pains in the rectum for two years past, which at length became insupportable, especially on going to stool. She entered the hospital on the 8th July, 1811. Clysters, baths, and potions were tried, without effect. The anus appeared to be in a natural state; but, when I wished to pass my finger into the rectum, I experienced a strong resistance, and I occasioned great pain. I felt, on the right side of the gut, and a little posteriorly, a fissure and a hard excrescence. The torments I caused were dreadful, on which account I abridged my research.

On the 2d of August, the patient had an injection early in the morning; at nine, I cut through each side of the sphincter. Five days afterwards, the patient voided a stool without suffering, and the largest tents now occasioned no pain.

On the 16th of September, the patient left the hospital cured.

2

CASE IV.—Louise Richeraud, twenty-one years of age, suffered for three months acute pains in going to stool, although clysters were administered for the purpose of facilitating the evacuation. The excrements were hard and small.

Local bleedings and suppositories had been employed without effect. The anus was strongly contracted, and cleft at the left side, a little posteriorly. I cut down on the fissure itself. During the seven first days which followed the operation, a retention of urine obliged us to have recourse to the catheter. At the end of twenty-two days, all treatment ceased, the largest penetrating into the anus without pain, and the patient went freely to stool.

CASE V.—A female, twenty-six years of age, had always enjoyed good health, when a coach, in which she was travelling to Orleans, was overturned. She received no contusion, but was greatly terrified. This was followed by nervous symptoms. Soon afterwards, hemorrhoids and obstinate constipation took place. When the bowels became again open, the evacuations were daily more painful, and were accompanied by acute pain, notwithstanding the clysters, vapour baths, laxatives, and other remedies employed. Tents were also used without effect. Her medical attendant, having seen a fissure on the right side of the anus, suspected a venereal cause, and recommended the employment of mercury.

The patient in terror consulted me. She was emaciated, pale, and weak; unable to leave her house; took nothing but liquid aliment, and her rest was almost entirely destroyed. The introduction of the finger into the anus occasioned cruel pain, which was prolonged for several hours, and accompanied by piercing cries.

I made an incision through the fissure and the sphincter. During the inflammation of the wound, some pains were caused by the introduction of the tents, but they finally disappeared, to return no more.

CASE VI.—A female, fifty-six years of age, during convalescence from malignant fever, had an obstinate constipation for the space of two months. The feces, large as chesnuts and very hard, required the greatest effort for their expulsion, although clysters and mild laxatives were not neglected. Gradually the evacuations became more difficult, were preceded and accompanied by acute pain, which lasted many hours, and left the patient during a part of the day in a state of profound stupor. Sleep at night was often interrupted by convulsive movements. The patient, having learnt by experience how dangerous it was to pass a single day without an evacuation, had renounced all solid aliment and wine, and lived on fresh broths, potages, and milk. Many practitioners recommended the use of tents, which she could not support, however slender. A suppository of elastic gum was also useless. These means aggravated the symptoms.

After four years of torment, she came to consult me. I recognised a fissure on the right side of the anus, and a spasm of the sphincter. I made the incision on the fissure itself, and soon the patient was enabled to go to stool without pain. For five years she has continued to enjoy good health.

Case VII.—In the month of December, 1809, I was consulted by M. N., who had, on the left side of the anus anteriorly, a superficial fissure, accompanied by spasmodic constriction; fifteen years previously he had gone to Geneva, and, after an excess in eating and drinking, he experienced constipation and difficulty of passing his urine; which long resisted the use of baths, fomentations, clysters, and bleeding. The hemorrhoidal veins swelled, with wandering pains in the limbs. Leeches mitigated the pain, and disgorged the distended vessels; but the pains soon became permanent, especially on evacuating the bowels, however soft the excreted matter.

Several practitioners counselled the strictest regimen, relaxing drinks, and tents; but all was ineffectual. When I operated, the coarctation of the anus was very great; the evacuations extremely painful. The incision was made on the fissure itself, was followed by no untoward symptom, and the result was most satisfactory.

Case VIII.—M. P——, of Chartres, aged forty-one years, had a fissure of the anus, with spasmodic stricture of the sphincter. Too much debilitated, when I operated, to give me all the particulars that I desired, the following were sent to me in writing several months after he was cured:

From the age of eighteen years I had hemorrhoids; my fundament has always been narrow, and the evacuation of feces difficult. In 1806, I had a severe illness. My hemorrhoids became very painful: one of them appeared at the exterior of the anus. Bleedings did not calm me. I experienced, in going to the water-closet, the most acute pain. In the efforts which I was compelled to make, a laceration to the extent of six lines was formed in the anus. Since this period the evil has always been on the increase. I passed seventeen days without sleeping. I lived on chicken broth, grapes and pears, to avoid increasing the excrement. At the end of a month my pains abated; but the orifice of the anus was scarcely large enough to receive the end of a canula. Towards the end of 1808, I went to Paris, for the purpose of consulting Mr. ———. My countenance was jaundiced, and liver affected. He prescribed an aperient drink and the application of twenty leeches. The anus was at that time almost free from pain; but, in the spring of 1809, the pains recurred with more force. I returned to Paris, to consult the same surgeon. He examined the anus, and ordered tents, which I did not apply. I consulted another, who prescribed laxatives and "douches ascendantes." I employed the latter, but the second application caused such acute pain that I could not support it. I then had recourse to you, sir, and the following is a copy of your written answer.

"There exists on the left part of the margin of the anus an hemorrhoidal excrescence, divided into two parts. Between the two portions is a fissure, which extends into the anus for the space of about six lines. The pain which the patient experiences at the time of going to stool, and afterwards, depends solely on the fissure and on the spasmodic constriction of the sphincter. I have frequently observed this malady, and I have discovered no other means of curing it than by making an incision, thereby converting the fissure into a simple wound, which destroys the constriction of the sphincter, and enables it readily to yield to the passage of the excrement."

I showed this opinion to M. Pinel, who was entirely of your advice. You know what I experienced at the time of the operation. My health at this moment is good. I have no pain in the anus; no difficulty in going to stool; no hemorrhoids; a little heat only when I have taken too much exercise: I then take a clyster, and all disappears. I frequently hunt; I am free from ennui, and my health was never better.

*Cases of Constriction, without Fissure.*

Case IX.—Madame de ——— from her childhood complained of extreme difficulty in the expulsion of feces. Ascribing this phenomenon to a heated temperament, her parents paid little attention to it, administering occasional clysters, and thus several years passed away. Between the ages of fifteen and sixteen, Madame de ——— could not evacuate the bowels without the aid of injections; and even then the discharge was accompanied and followed by great pain.

A feeling of laceration and pricking caused a suspicion of hemorrhoids. The pains, which at first were supportable and of slight duration, became gradually more acute, and continued fifteen or sixteen hours after every evacuation.

All the physicians and surgeons of the country where Madame de ——— then resided were consulted; those of Havre and Rouen also. One of them introduced, for the space of three months, pledgets of lint covered with cerate.

At length the increasing sufferings of the patient brought her to Paris. She consulted Desault, Vicq d'Azyr, Sabatier, Portal, and Chossart. The latter employed tents for several months. The pains became agonising, and the anus constricted to such a degree as no longer to admit the pipe of a syringe. She then had recourse to purgatives every other day; but this produced extreme emaciation and increased pain during the evacuation.

Worn out by the treatment, and losing all hope of being cured, in consequence of an indiscreet assertion of Desault that she would die if she could not bear the tents, she renounced the succour of medicine for the space of four years. At the solicitations, however, of her friends, she at length consented to have recourse to me. I made two lateral incisions on the sphincters of the anus, and kept the lips of the wound apart by tents. The cicatrisation was prompt, and the evacuations became easy. The pains entirely ceased, and the patient recovered her spirits and a healthy appearance.

CASE X.—Laurent Cistern experienced, at the age of thirty-one years, after long constipation, acute pains in the anus, which efforts to go to stool rendered agonising. From this moment the alvine evacuations could not be passed without inexpressible anguish, which continued for four or five hours. When he was up or in bed he suffered little, but when seated the pains were more acute; on which account he quitted the trade of a shoemaker. During thirty months laxatives were the only remedies which produced relief.

On the 26th of November, 1809, he entered the Hôpital la Charité. I discovered, at the right side of the inner part of the anus, a hard, thick, and painful callous point. This point was the principal seat of the pain which was experienced on going to stool. The sphincter contracted strongly on my finger, especially when I pressed upon this hard point. I put the patient on low diet, and prescribed the use of diluents and a gentle purgative. On the following day a clyster was administered; and, on the third, I cut the sphincter through the hard, thickened, and painful point of which I have spoken. The wound slowly cicatrised. During some time the fecal matters occasioned obscure pains in passing over the wound, and afterwards over the cicatrix. But the pains ceased, and many months after his exit from the hospital he came to see us, and was perfectly cured.

*Case of Constriction, where the existence of Fissure was doubtful.*

CASE XI.—Alexis Cuby, fifty-two years of age, has experienced, for two years and a half past, pains in going to stool. These, which were slight and only felt at intervals at the commencement, became so acute that the patient compared them to a red-hot iron introduced into the rectum. The use of injections prepared with narcotic substances procured but transitory relief. All other remedies were equally unsuccessful.

When he consulted me, I told him that an operation alone would put an end to his sufferings. The anus was so contracted that my finger penetrated with difficulty, and caused intense pain. Towards the left side, where the most acute pain was experienced, I *thought* I felt a fissure: I cut the sphincter at this part, and applied tents for the space of forty-one days: during the latter month of this period he went to stool without experiencing the smallest pain.

M. Boyer says that he has never seen the disease in children or adolescents: it may therefore be important to add the case of an infant, which occurred in his own hospital. It is reported in the Dictionnaire des Sciences Medicales.

Case XII.—A child, three or four days old, was brought to the Hôpital la Charité by its nurse, from whom we learnt that it had voided no excrement. The belly was tense and painful, and the child cried continually. The anus, on examination, was found to be extremely constricted, so that a probe could scarcely be passed into it. The sphincter was divided in the direction of the os coccygis. Meconium and feces came away immediately in abundance, which so relieved the child that it ceased to cry, and was perfectly cured.

---

